# Quantum Dots Do Not Alter the Differentiation Potential of Pancreatic Stem Cells and Are Distributed Randomly among Daughter Cells

**DOI:** 10.1155/2013/918242

**Published:** 2013-07-24

**Authors:** S. Danner, H. Benzin, T. Vollbrandt, J. Oder, A. Richter, C. Kruse

**Affiliations:** ^1^Fraunhofer Research Institution for Marine Biotechnology, 23562 Luebeck, Germany; ^2^Core Facility Cell Sorting, University Medical Center Schleswig-Holstein, 23538 Luebeck, Germany

## Abstract

With the increasing relevance of cell-based therapies, there is a demand for cell-labeling techniques for *in vitro* and *in vivo* studies. For the reasonable tracking of transplanted stem cells in animal models, the usage of quantum dots (QDs) for sensitive cellular imaging has major advances. QDs could be delivered to the cytoplasm of the cells providing intense and stable fluorescence. Although QDs are emerging as favourable nanoparticles for bioimaging, substantial investigations are still required to consider their application for adult stem cells. Therefore, rat pancreatic stem cells (PSCs) were labeled with different concentrations of CdSe quantum dots (Qtracker 605 nanocrystals). The QD labeled PSCs showed normal proliferation and their usual spontaneous differentiation potential *in vitro*. The labeling of the cell population was concentration dependent, with increasing cell load from 5 nM QDs to 20 nM QDs. With time-lapse microscopy, we observed that the transmission of the QD particles during cell divisions was random, appearing as equal or unequal transmission to daughter cells. We report here that QDs offered an efficient and nontoxic way to label pancreatic stem cells without genetic modifications. In summary, QD nanocrystals are a promising tool for stem cell labeling and facilitate tracking of transplanted cells in animal models.

## 1. Introduction

Adult stem cells derived from the pancreas have a remarkable potential for self-renewal and multilineage differentiation [1–4]. Recently, we and other groups have reported on the isolation and propagation of similar adult stem cell populations from salivary glands and sweat glands [5–8]. Using simple isolation procedures, exocrine glands serve as promising source for cell populations displaying all essential characteristics of multipotent stem cells *in vitro*. These glandular stem cells have already been analyzed for their regeneration potential in preclinical studies [9, 10]. The benefit of a cell-based therapy has been exemplarily shown for pancreatic stem cell populations in rodent models. Both mouse pancreatic stem cells [9] and rat pancreatic stem cells [10] accelerated wound healing of full-thickness skin defects and enhanced vascularization when seeded onto a collagen scaffold for dermal regeneration.

However, prior to application of stem cell populations in regenerative medicine, major challenges remain to be overcome. The exploration of the cells' survival, proliferation and possible differentiation in animal models requires a cell label to trace transplanted cells *in vivo*. Current methods for labeling adult stem cells include iron particles [11–13], organic fluorescent dyes [14], or fluorescent proteins expressed by the cells [15]. 

Recent developments in nanotechnology have provided promising and efficient tools for biomedical applications and regenerative medicine. Particularly, fluorescent nanoparticles like quantum dots (QDs) could be useful for manipulating and tracking stem cells. QDs are characterized by narrow band emission and broadband excitation. They have remarkable advantages due to their photostability and durable fluorescence intensity which improves long-term cell labeling and implies advanced performance compared to organic dyes and fluorescent proteins [16, 17]. QDs have excitation and emission spectra that are different from the body's autofluorescence allowing distinct and reliable tracking of transplanted cells [18]. Quantum dots are inorganic nanocrystals and have a typically size range of 2–10 nm. They are composed of a semiconductor core (e.g., CdSe, CdTe) encased within a shell comprised of a second semiconductor material (e.g., ZnS) [19]. To generate a biological surface for uptake into the cells the QDs had to be coated with a variable, biocompatible outer layer. In most cases the internalization of QDs is achieved by the use of certain peptides such as cholera toxin [20], TAT-peptide [21, 22], RGD [23, 24] or phospholipids [25]. QDs have been already used to label different kinds of cells *in vitro* including tumor cells [26], endothelial cells [27], fibroblasts [28], keratinocytes [29], mesenchymal stem cells [30, 31] and embryonic stem cells [32]. Their advantageous properties offered also many strategies *in vivo* including labeling of zebrafish embryos [33] or Xenopus embryos [34]. Whereas several reports suggest that QDs are non-toxic [26, 34, 35] some groups reveal possible cytotoxic or aberrant effects of QDs depending on their size, coating and physiochemical properties [29, 36, 37]. For example, it has been shown that human bone marrow mesenchymal stem cells were affected in their osteogenic differentiation *in vitro* by CdSe/ZnS quantum dot labels [36].

In order to address above questions we labeled rat pancreatic stem cells with different concentrations of Qdot 605 nanocrystals. These QDs have a cadmium selenium core and a zinc sulfide outer shell. They have a diameter of 5–15 nm and after coating them with a targeting polyarginine peptide they are endocytosed by the cells [38, 39]. We quantified the cellular total QD load by FACS, determined viability and proliferation and analysed the differentiation potential by real-time PCR and immunocytochemistry. In addition, the distribution of QDs among daughter cells was determined by time-lapse microscopy.

## 2. Materials and Methods

### 2.1. Cell Culture

Rat pancreatic stem cells were cultivated after isolation described by Kruse et al., 2006 [2] using DMEM (Gibco Invitrogen, Germany) with 10% (v/v) fetal calf serum (FCS) (PAA, Austria) and Penicillin/Streptomycin (PAA, Austria) at 37°C and 5% CO_2_. When full confluency on the cell culture plastics (TPP, Switzerland) was reached, the subcultivation was performed after washing with PBS (Gibco Invitrogen, Germany) by incubation with 0,05% Trypsin (PAA, Austria) for 2 minutes at 37°C. The reaction was stopped with double amount of media followed by a centrifugation for 5 minutes at 180 g. After resuspending the pellet with media a reseeding of the cells was performed in a ratio of 1 : 3. For long term preservation cells are frozen in a cryo media containing 90% FCS and 10% DMSO (Carl Roth, Germany) for a minimum of 24 hours in an isopropanol-coated box followed by a transfer to liquid nitrogen. Thawing of the cells was performed by fast resuspendation in media and centrifugation for 5 minutes with 180 g. Subsequently, they were reseeded as described above on the same growth area as they were cultured before and cultivated for at least one passage. For continuous supply with nutrients and removal of metabolites, the media was completely changed every third day.

### 2.2. Labeling Procedure

The labeling with QD nanocrystals, namely, Qtracker 605 Cell Labeling Kit (Invitrogen Molecular Probes, Germany), was performed according to the manufacturer's protocol. Briefly, we mixed component A with B in equal ratios, incubated for 5 minutes at room temperature and added the sufficient amount of cultivation media for each concentration. This suspension was then supplied to the cells and incubated for 1 hour at 37°C and 5% CO_2_. We tested three different concentrations—the recommended 10 nM suspension, as well as 5 nM and 20 nM. Finally, the cells were washed twice with media and propagated until analysis with the above described media.

### 2.3. Cell Counting and Growth Curve

Cell counting was performed using a NucleoCounter (Chemometec, Denmark) and the associated reagents. Briefly, during subcultivation an aliquot of 50 *μ*L of the cell suspension was mixed with the same amount of lysis and stabilization buffer (both from Chemometec, Denmark). The suspension was then absorbed by a NucleoCounter's cassette according to the user's manual and the instrument measured the number of propidium-iodide labeled nuclei in the sample. 

For the initial analysis of an influence of QDs on cell growth, we measured the cell count of labeled and unlabeled cells in one passage on days 1, 3, 5, and 7. The labeling procedure was performed in a flask with confluent stem cells and with 20 nM QDs. After three hours, cells were trypsinized and seeded at a density of 90 000 cells in six well plates. Mean cell counts on each day were calculated from triplets. 

In one experiment, we also analysed the QD effect on long-term proliferation. The growth curves of both, unlabeled and QD labeled populations, were started with an adjusted cell count of one million cells for each population. Over a period of 16 days, the cells were subcultivated in a ratio of 1 : 3 for 4 times. The cell number of the populations at each subcultivation was determined as described before and mathematically extrapolated for the presentation of the proliferation as a graph.

### 2.4. Flow Cytometry

For fluorescent activated cell sorting analysis QD labeled (5 nM, 10 nM, and 20 nM) and unlabeled cells as a control were seeded in 25 cm^2^ culture flasks. At the time of analysis (24 h, 48 h, and 96 h after labeling), the cells were trypsinized, counted and pelleted according to the previously described cultivation protocol. Subsequently, they were resuspended in ice-cold FACS-buffer containing 2% FCS (v/v) diluted in PBS. The measurement of cell aggregates was prevented by the use of a 50 *μ*m filter (Partec, Germany). The samples were stored on ice and mixed before analysis. The immediate measurement of the fluorescence intensities was performed on a MoFlo high-performance cell sorter (Beckman Coulter). MoFlo was operated with a 70 *μ*m nozzle at 60 Psi. QDs were excited using a Coherent Innova 90 C argon laser tuned to MLUV 50 mW. Data were analyzed using Summit 4.3 software.

### 2.5. Time Lapse

To observe the cell's behaviour after labeling and the transfer of QDs within the cell population, the cells were treated with the described QDs concentrations (5 nM, 10 nM, and 20 nM) and cultivated on a microscope with incubation chamber (Zeiss MicroImaging, Germany) at 37°C and 5% CO_2_ over a period of 100 hours. An untreated cell population was used as control. At the beginning approximately 5.000 cells per cm^2^ were seeded into 6-well cell culture plates (TPP, Switzerland). The labeling was performed 24 h later and subsequently the samples were monitored using a time-lapse microscope. A picture was taken at defined positions in each sample every 15 minutes using the following settings: 10-fold magnification, excitation wavelength: 555 nm, numeric aperture: 0.45/Ph1, and reflector block: 43HE. Finally, the time series of pictures was analyzed. The observed ways of QD transfer during cell divisions (unequal and equal) were quantified by analyzing time lapse picture series of four experiments. In total, we estimated 242 cell divisions and prorated them to the equal and unequal way of QD transmission.

### 2.6. Immunocytochemistry

For immunocytochemical stainings, 7.500 cells per cm^2^, both QD labeled (20 nM) and unlabeled, were seeded into 2-well Culture Slides (BD, Germany) and cultivated for 2 days. Prior fixation with 4% (w/v) paraformaldehyde (Merck, Germany) and with 10% (w/v) sucrose (Roth, Germany) the cells were washed once with PBS (Gibco Invitrogen, Germany), and then incubated 15 minutes with the fixation solution. After three times washing with PBS, unspecific binding sites were saturated by treatment with 1.7% (v/v) normal goat serum (Vector Laboratories, USA) in PBS for at least 20 minutes at room temperature. Afterwards, primary antibodies were diluted in TBST buffer containing 0.1% BSA (PAA, Austria) and incubated with the cells in a humid chamber for 1 h at 37°C. The primary antisera were directed against amylase (mouse monoclonal, 1 : 50, Santa Cruz, USA); GATA-4 (rabbit polyclonal, 1 : 100, Santa Cruz, USA); Ki67 (rabbit polyclonal, 1 : 500, Abcam, UK); nanog (rabbit polyclonal, 1 : 1000, Millipore, USA); nestin (mouse monoclonal, 1 : 100, Chemicon, Switzerland), neurofilaments light, medium heavy, and heavy chains (1 : 1 : 1 mixture, rabbit polyclonal, 1 : 500, Serotec, UK); nucleostemin (rabbit polyclonal, 1 : 100, Santa Cruz, USA); vimentin (mouse monoclonal, 1 : 500, DAKO, Germany), and *α*-smooth muscle actin (*α*-SMA, mouse monoclonal, 1 : 100, DAKO, Denmark). After rinsing three times with PBS, the slides were exposed to FITC-labeled secondary antibodies, both anti-mouse IgG and anti-rabbit IgG (1 : 200, Jackson ImmunoResearch Europe, UK) and diluted in PBS under the same conditions. Following secondary antibody incubation, the cells were washed three times with PBS, once with distilled water and mounted with DAPI-containing Vectashield Mounting Medium (Vector Laboratories, USA). The differentiation potential of the cells was analysed using a fluorescence microscope Axioskop 2 and a confocal laser scanning microscope LSM710 (both from Carl Zeiss, Germany). The negative controls were carried out using only the secondary antibodies and showed only a nonspecific, faint staining (data not shown).

### 2.7. PCR

Total RNA from QD labeled and unlabeled cells grown confluent in 75 cm^2^ culture flasks was isolated using the RNeasy Plus Mini kit (Qiagen, Germany) according to the manufacturer's instructions. RNA concentrations were determined by absorption measurement at 260 nm using a nanodrop spectrophotometer (Peqlab, Germany). Reverse transcription including a digestion step of genomic DNA was carried out using the QuantiTect Reverse Transcription kit (Qiagen, Germany). For each sample, cDNA was synthesized from 1 *μ*g total RNA. Real-time PCR was performed with 1 *μ*L cDNA in a 25 *μ*L reaction volume using the QuantiFast SYBR Green PCR kit and rat QuantiTect Primers (both from Qiagen, Germany) presented in Table 1 in a RealPlex2-Mastercycler (Eppendorf, Germany). The fluorescence threshold value was ascertained using the CalQplex algorithm from the Mastercycler ep realplex software (Eppendorf, Germany). Relative transcriptional activity was determined by the ΔΔct method with *β*-actin gene expression as an endogenous reference. 

## 3. Results

### 3.1. Labeling of Pancreatic Stem Cells with Different QD Concentrations

**Figure 1:**
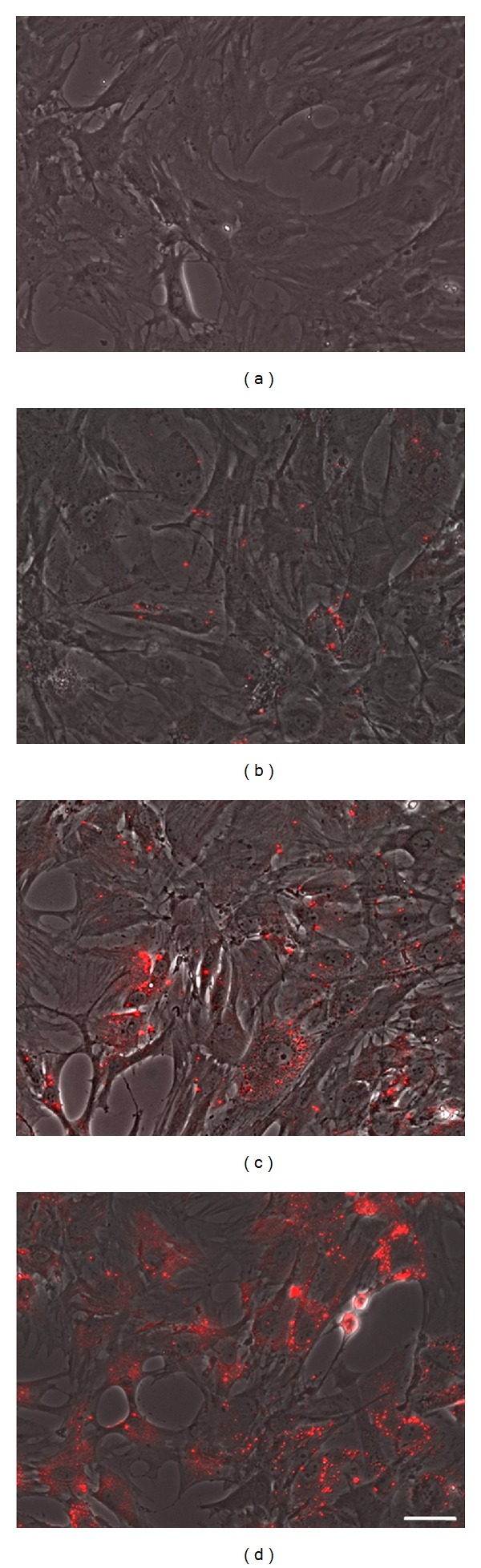
Fluorescence intensity and distribution of QD treated living stem cells 24 h after labeling. (a) Control without QDs, (b) 5 nM QDs, (c) 10 nM QDs, and (d) 20 nM QDs. Scale bar represents 50 *μ*m.

In the first attempt, we analyzed the optimal quantum dot labeling concentration to achieve a complete and homogenous nanoparticle distribution within the stem cell population. Pancreatic stem cells were therefore treated with the manufacturer's proposal of 10 nM and also with the half (5 nM) and double (20 nM) concentration.  Figure 1 shows fluorescent microscopic images of the cell layer 24 h after labeling. Obviously, the cells have incorporated the quantum dots and the labeling intensity is concentration dependent. The QD distribution within each cell is not homogenous but rather in aggregates. Only in the 10 nM and 20 nM quantum dot label solutions are enough particles to label each cell of the cell layer. Labeling with the 5 nM QD concentration resulted in a weak fluorescent tag in only a few cells. 

### 3.2. Localization of Nanoparticles within the Cells

**Figure 2:**
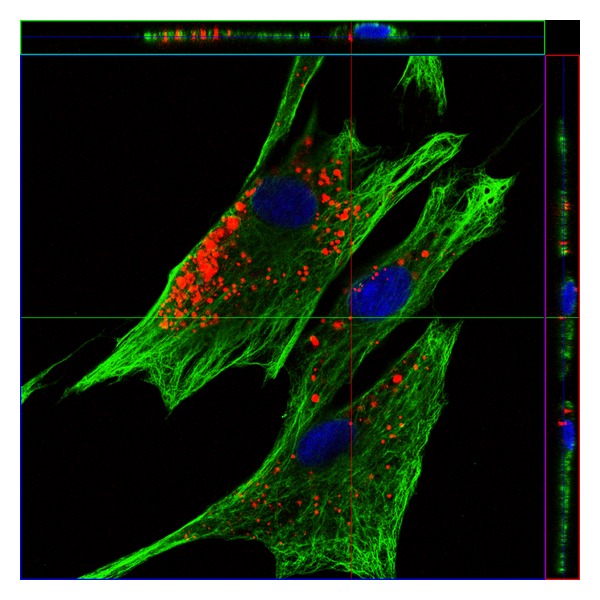
Spatial distribution of QDs (red) within the cells. Confocal image of labeled PSCs immunostained for Vimentin (green). Nuclei were stained with DAPI (blue).

The arrangement of quantum dots within the cell bodies was furthermore analyzed in detail by confocal microscopy. By immunostaining the cells with a vimentin antibody after QD labeling, we can visualize the cytoskeleton of the cells and can analyze the spatial distribution of the QDs after creating Z-Stacks with the microscopic software. This 3D image (Figure 2) confirmed the intracellular accumulation of QD aggregates within the cell cytoplasm dispersed between the vimentin filaments. QD aggregates were often localized surrounding the nucleus and in the majority of analyzed cells these aggregates do not enter the nucleus.

### 3.3. FACS Analysis of QDs Cell Load and Retention

**Figure 3:**
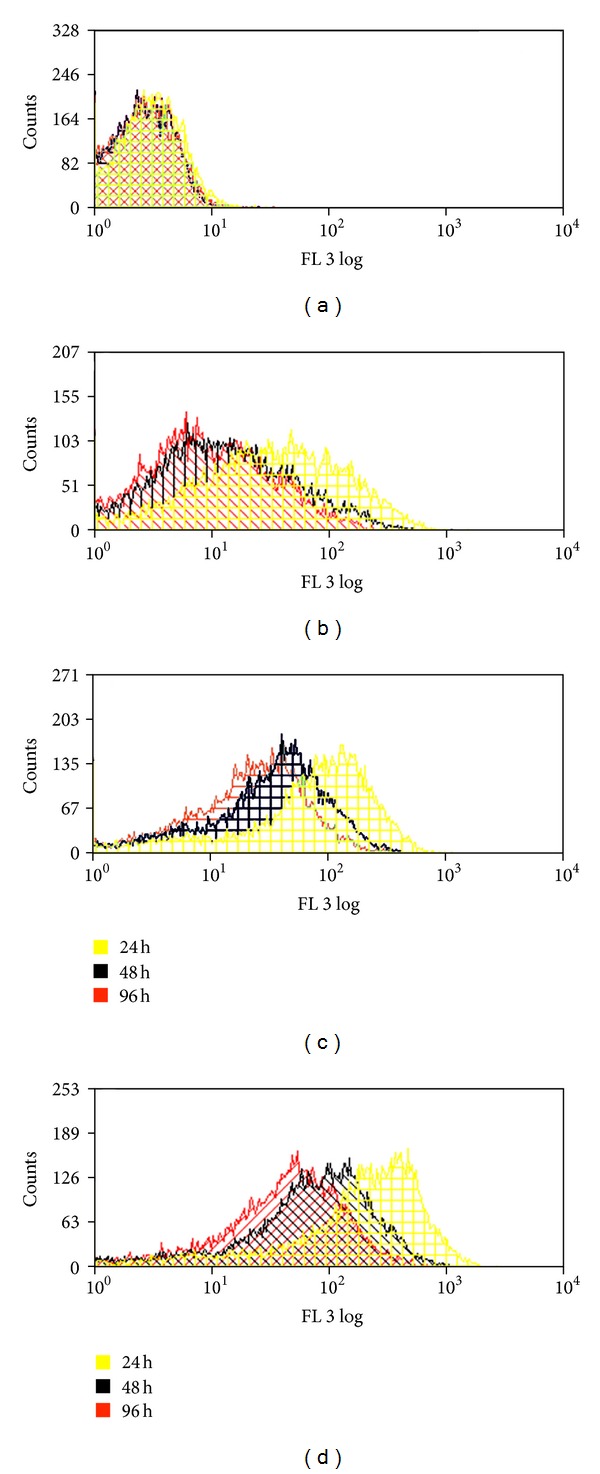
FACS analysis of QDs cell load over cultivation time. The fluorescence per cell was analysed after 24 h, 48 h, and 96 h. (a) Control cells without QDs, (b) cells labeled with 5 nM QDs, (c) cells labeled with 10 nM QDs, and (d) cells labelled with 20 nM QDs.

**Figure 4:**
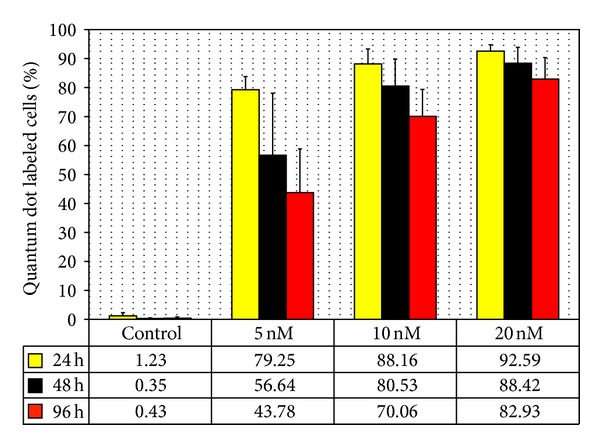
Quantitative analysis of QD labeled cells measured by FACS analysis. The amount of QD labeled cells was estimated for three label concentrations (5 nM, 10 nM, and 20 nM) and for three time points (24 h, 48 h, and 96 h) (*n* = 2). Total count 10.000 cells.

During expansion of PSC in *in vitro* cultures, we analyzed the cells' QD load and the retention of the label after 24 h, 48 h, and 96 h by FACS analysis. The three different QD concentrations of 5 nM, 10 nM, and 20 nM have an influence on the effectiveness of the label procedure and the QD retention within the proliferating cell population. The distribution of the measured fluorescence per cell varies effecting a bell-shaped curve in the FACS profile (Figure 3). All the distribution curves shift to lower fluorescence intensities during time, so that the all over fluorescent intensity of the population is the lowest after 96 h. This effect is more prominent with decreasing initial label concentrations. A quantitative evaluation of QD positive cells within the PSC population shows the vanishing of the label over time (Figure 4). 24 h after the labeling procedure, the best labeling success is achieved with the 20 nM QD concentration (92,59% ± 2,2%), where over 90% of the cells were counted as fluorescently labeled. This amount decreases to 82,93% ± 7,4% after 96 h of culture. This loss of QD label is pronounced in the stem cell cultures labeled with lower concentrations. For the 5 nM concentration only an amount of 79,25% ± 4,5% of the stem cells is initially labeled and this amount decreased to 43,78% ± 15,1% after *in vitro* propagation for 96 h. Based on these results, we decided to use the 20 nM QD concentration as standard and to evaluate further cell parameters in regard to this nanoparticle concentration.

### 3.4. Time Lapse Analysis of QD Distribution during Cell Division

**Figure 5:**
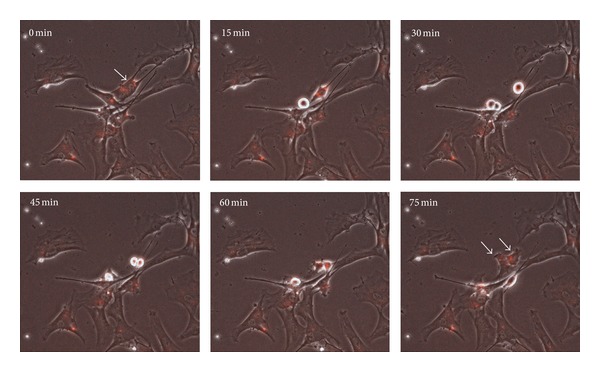
Time-lapse study showing equal passing on of QDs during cell division (see arrows). Selected microscopy images acquired during cultivation of labeled cells show a time period of 75 min. Overlay images were created from the fluorescent and the phase contrast image.

**Figure 6:**
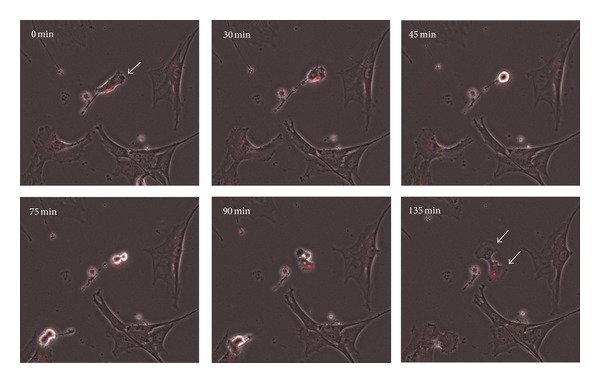
Time-lapse study showing unequal passing on of QDs during cell division (see arrows). Selected microscopy images acquired during cultivation of labeled cells show a time period of 135 min. Overlay images were created from the fluorescent and the phase contrast image.

**Figure 7:**
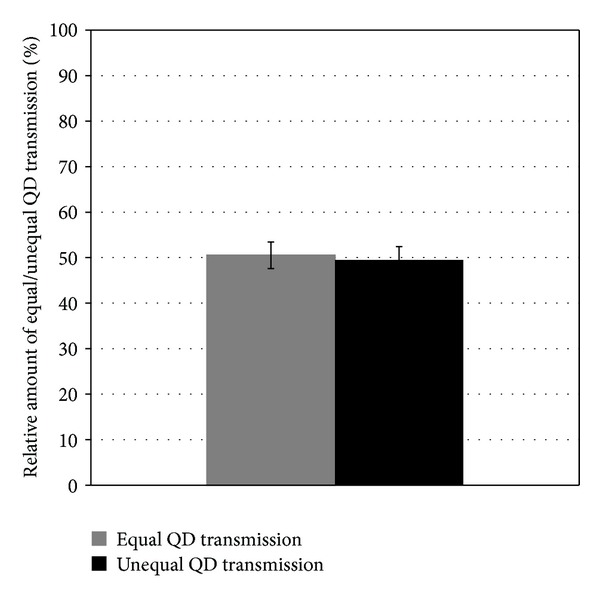
Distribution of equal and unequal cell divisions in QD labeled cell populations. The relative amount of cell divisions with equally and unequally transferred QDs was quantified by analyzing time lapse picture series of four experiments. In total, 242 cell divisions were prorated to the equal and unequal way of QD transmission.

In order to analyze the passing on of QDs in expanding stem cell cultures, we observed the labeled cell population with time lapse microscopy. Here we were able to follow the distribution of nanoparticle aggregates during cell division. Obviously the QDs were passed to the daughter cells, but in two different ways. There are examples for an equal transfer to the two cells of the next generation (Figure 5) as well as conditions where the nanoparticles were only present in one daughter cell after cell division (Figure 6). This unequal distribution of QDs was no rare event, so both possibilities to pass on the QDs during expansion of the cell population occur in parallel. In order to quantify the extend of equal and unequal QD transmission, we analyzed four time lapse picture series and assign 242 cell divisions to the equal or unequal transmission way. As demonstrated with the statistically estimation of Figure 7, there was no preferred way of QD transmission and both ways occur with nearly the same frequency.

### 3.5. Proliferation Characteristics of QD Labeled Cells

**Figure 8:**
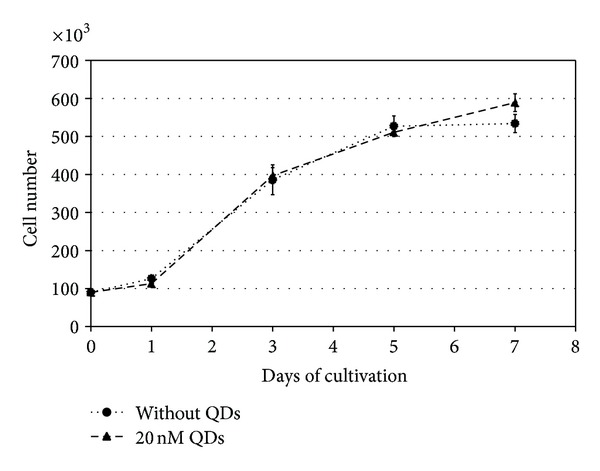
Growth curve of QD labeled and unlabeled stem cell populations during 7 days of cultivation. Cells were counted on days 1, 3, 5, and 7. Mean cell count was calculated from three experiments.

**Figure 9:**
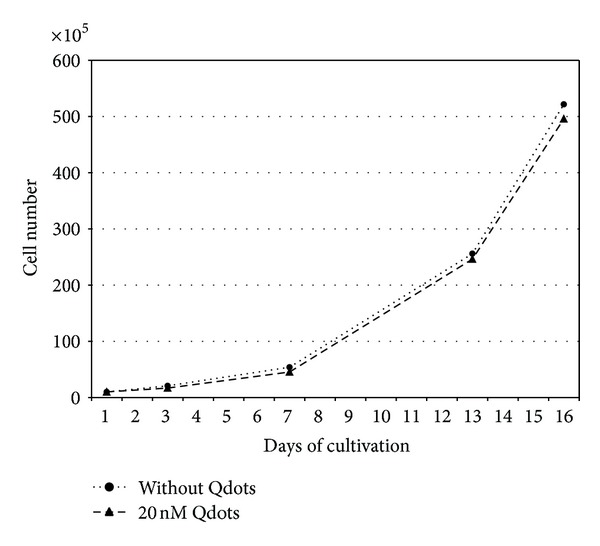
Long-term proliferation of a QD labeled and an unlabeled stem cell population during subcultivation within a period of 16 days. The cells were passaged and counted on days 3, 7, 13, and 16. Curve was calculated from one experiment.

To study the influence of QDs during *in vitro* growth of PSCs, we performed growth curves within a cultivation period of 7 days. During this short-term cultivation, there was no obvious effect on the proliferation capacity of PSCs (Figure 8). In one experiment, we also analyzed the impact of QD labeling on long-term proliferation. During subcultivation of PSCs over four passages, we measured the cell count of labeled and unlabeled stem cells. The uptake of QDs even in a concentration of 20 nM had no adverse effect on the cell population during the following cultivation phase of 16 days. The growth curves of QD labeled and unlabeled stem cell populations were almost identical (Figure 9).

### 3.6. Effect of QD Labeling on the Differentiation Capabilities of Pancreatic Stem Cells

Pancreatic stem cells are known to have a multipotent differentiation potential *in vitro*. They express spontaneously a subset of different mRNAs and proteins, indicating differentiation pathways to endodermal, mesodermal, and ectodermal cell lineages. We here investigated the impact of QD labeling on these stem cell properties on the transcriptional and translational level performing a quantitative reverse transcription PCR as well as immunocytochemical stainings.

**Figure 10:**
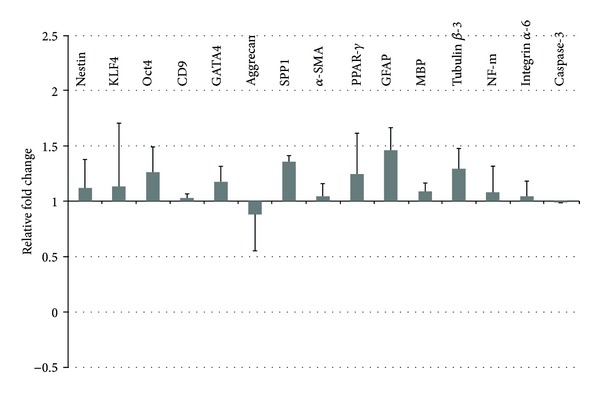
The transcriptional activity of QD labeled stem cells was analyzed by quantitative PCR and set in relation to untreated control approaches (representing the value “1”). Changes in gene expression are displayed as upregulation (>1) and downregulation (<1). Standard deviations were calculated from two experiments. Transcripts related to stem cells (Nestin, KLF4, Oct4, CD9), endodermal cells (GATA4), mesodermal cells (Aggrecan, SPP1, *α*-SMA, PPAR-*γ*), and ectodermal cells (GFAP, Tubulin *β*-3, NF) were analyzed. Also the mRNA expression level of the surface protein Integrin alpha-6 and the apoptosis marker caspase-3 was estimated.

The gene expression profile of unlabeled cells was compared to that of QD labeled cells by normalizing the data against the unlabeled cell samples. The gene expression analysis of various transcripts revealed only minor changes for the analyzed stem cell-related and differentiation-associated genes (Figure 10). The relative mRNA expression of all analyzed genes fluctuates around 1. For an induced up or down regulation of transcription, one would estimate a twofold change at least (relative expression of >2 or <0,5). Comparing the labeled cell populations with the unlabeled, we cannot observe expression changes in this magnitude, indicating no gross impact of QD labeling. Interestingly, even the apoptosis marker caspase-3 shows no upregulation in QD labeled stem cell populations. 

**Figure 11:**
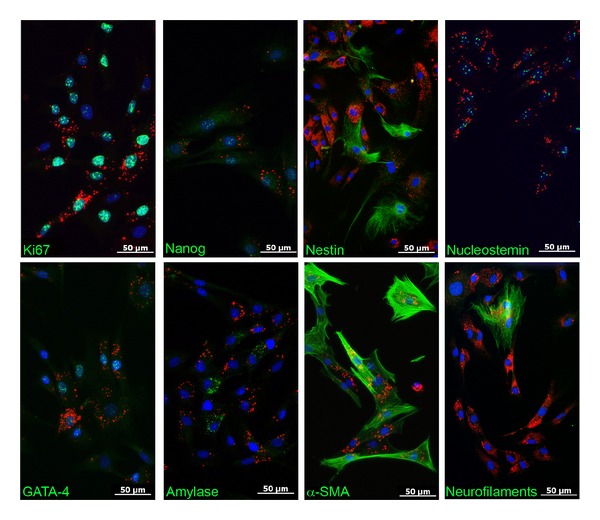
Immunocytochemical localization of stem cell and lineage specific proteins (green) in QD labeled (red) stem cells. QD labeled cells stained positive for nuclear (Ki67, Nanog, Nucleostemin, GATA-4), secretory (Amylase), and filamentous proteins (Nestin, *α*-SMA, Neurofilaments) indicating spontaneous *in vitro* differentiation. Nuclei were stained with DAPI (blue).

These data were confirmed by analyzing the cells protein expression profile. We carried out immunocytochemical staining of the QD labeled stem cell population and analyzed the expression of specific proteins related to stem cells (Ki67, nanog, nestin, nucleostemin) and differentiated cells (endoderm: GATA4, amylase; mesoderm: *α*-SMA; ectoderm: neurofilaments). For all the analyzed proteins, we detect double labeling for the specific protein and the fluorescent nanoparticles (Figure 11). Thus, the multilineage differentiation potential of PSC during *in vitro* culture seems to be unaffected by QD labeling in regard to the analyzed markers.

## 4. Discussion

QD nanocrystals are an easy and efficient way to tag cells with an intense fluorescent label. Particularly transplantation studies with stem cells will profit from this tool, which enables tracking of single cells in an experimental animal. We have previously shown that transplanted rat pancreatic stem cells enhance the regeneration of full thickness skin defects in a mouse model [10]. This qualitative benefit has up to now not been analyzed in detail concerning fate and function of the delivered stem cells due to an absent label for tracking. In the present study, we explored the application of QD nanocrystals for labeling rat pancreatic stem cells *in vitro*. Depending on the QD concentration, we achieved progressive fluorescent intensities and throughout labeling with increasing concentrations of the label solution. The manufacturer's recommendation for the optimal concentration of 10 nM QDs properly labels the cells and even doubling of the QD concentration is well tolerated by PSCs. Confocal microscopy confirmed the localization of nanocrystal aggregates within the cell cytoplasm. Obviously, the QD nanocrystals do not or just marginally enter the nucleus. Lovrić et al. [40] have demonstrated toxic effects of quantum dots depending on their size and correlated the subcellular localization within or outside the nucleus with these effects. Interestingly, red emitting quantum dots stayed in the cytoplasm and are less cytotoxic than smaller green emitting ones, which accumulated in the nucleus [40]. In line with these observations, we monitored the subcellular distribution of the QDot 605 nanocrystal aggregates in the cytosol of rat pancreatic stem cells and corroborate thereby earlier observations for nanocrystals in rat and human mesenchymal stem cells [31, 37]. We assume that for cell tracking purposes the red emitting QDs are anyhow superior to the green emitting ones, as they are better distinguishable from tissue autofluorescence as well. 

By analyzing the stability of the label over culture time, we observed diminishing fluorescence intensities when cells are proliferating. The quantitative FACS analysis revealed that the percentage of labeled cells in the stem cell population decreased. This effect was less prominent in cell populations labeled with high QD concentrations, which retain an amount of fluorescently labeled cells of about 80% after 48 h. These observations have previously also been made in other experiments where mouse embryonic stem cells and embryonic fibroblasts were labeled with QD 655 nanocrystals [32]. Also for rat and human mesenchymal stem cells the loss of the fluorescent label over time and with progressive cell divisions had been observed and discussed [31, 37]. In order to identify the cause of this effect, we continuously monitored the QD labeled cell population by time-lapse microscopy. By following the passing on of QD aggregates to daughter cells during cell division, we discovered two ways of QD transfer: (I) equal transmission of QD aggregates to the daughter cells and (II) unequal transmission of QD during cell division. The second event resulted in two daughter cells with and without the fluorescent label and occurs in 50% of cell divisions. This phenomenon seems to be the cause for the progressive loss of the fluorescent label within the stem cell population with time. In line with these observations also asymmetric divisions of stem cells as well as nonequivalent distribution of nanoparticle-loaded endosomes between daughter cells might be a reason [38, 41]. In a study by Pi et al. [32], a quick loss of QD labeling in embryonic stem cells was observed which had been suggested to be due to the active excretion of QDs by membrane transporters. If PSCs have the ability to actively excrete QDs is currently unknown and has to be investigated in further analyses. With respect to these aspects we however suppose that the slight loss of fluorescently marked cells in the cultured cell populations under self-renewal conditions *in vitro* might be occur to a lesser extend when the cells were transplanted to tissues *in vivo*.

Besides the mechanisms of QD transfer or excretion, their cytotoxicity and the possibility of aberrant effects on gene expression remain a major concern. In the current work, the cytocompatibility of QDs during short-term and long-term proliferation has been compared in stem cells with and without QDs. As the growth curves of both cell populations were nearly congruent, no apparent deleterious effects could be discovered for the labeled cell population. Overall QDs seem to be well tolerated by proliferating cells and do not interfere with cell division, which could be also evidenced for red emitting QDs by other working groups [24, 32, 35, 37]. Owing to the special properties of stem cell populations, it is of major importance to warrant unaffected gene and protein expression and to characterize their multipotent differentiation potential with respect to any QD induced changes. Pancreatic stem cells exhibit a multilineage differentiation potential and form cells of endodermal, ectodermal, and mesodermal origin spontaneously *in vitro* [2, 42, 43]. By analyzing certain consistently expressed transcripts, we analyzed the gene expression profile of QD labeled and nonlabeled cells. The normalized relative expression levels oscillate around one and none of the analyzed genes was up- or downregulated more than twofold. This magnitude of alteration is minor as glandular stem cells can up or down regulate their gene expression, for example, due to a targeted differentiation approach more than tenfold [44]. Thus, with respect to the analyzed transcripts, we observed no apparent negative effect of QDs on the stem cells' gene expression, even the apoptosis-related transcript of caspase3 was not up regulated. In order to validate these findings also on the protein level, we characterized the expression of specific proteins by immunocytochemical stainings. With the gained results, we emphasize the cytofriendly features of QDs as the pancreatic stem cells accomplish proper spontaneous differentiation towards the specific cell types also when they contain QDs. Within the scope of the analyzed markers, none of the three differentiation pathways for ectodermal cells (neurofilament-positive), endodermal cells (amylase-positive, GATA4-positive), or mesodermal cells (*α*-SMA-positive) was impaired after QD labeling and the expression of all proliferation- and stem cell-associated marker proteins (Ki67, nanog, nestin, and nucleostemin) was unaltered as well. The effects of QDs on stem cell differentiation have been already evaluated for different stem cell types with varying results. Recently, Rak-Raszewska et al. [35] demonstrated that mouse embryonic stem cells as well as kidney stem cells are not affected in their behavior by red-emitting QD labels whereas Hsieh et al. [36] showed an influence of green-emitting QDs on the osteogenic differentiation capacity of human bone marrow stem cells. This differing result corroborates the cyto-friendly features of the larger red emitting QDs and the harmful impact of cell labels with the smaller green-emitting ones. Applying QDot 605 nanocrystals for the here analyzed pancreatic stem cells resulted in unchanged expression profiles for the investigated transcripts and proteins suggesting unaffected spontaneous differentiation during *in vitro* propagation. If there will be an effect of the QDs when these cell populations undergo, an induced differentiation towards certain cell types had not been determined and will be subject of further investigations. 

With several methods we have estimated the impact of QDs on *in vitro* proliferation and differentiation and in general we could not reveal any substantial influence of the labeling on PSCs. A possible usage of QDs as cell friendly label should however be further estimated in animal experiments, since there are additional parameters to be considered (e.g., metabolism, toxicology, excretion). 

## 5. Conclusion

The present study investigates the feasibility of labeling pancreatic stem cells with QDs to facilitate cell tracking of transplanted cells in animal experiments. We have demonstrated that PSCs could be efficiently labeled by QDs without impact on cell viability or proliferation. With regard to the analyzed genes and proteins, we furthermore could warrant no apparent deleterious effects on the cells spontaneous differentiation potential *in vitro*. When cultured under self-renewal conditions *in vitro,* there is a moderate loss of the fluorescent QD label due to repeated cell divisions. The transmission of the QD particles during cell divisions could be equal or unequal resulting in QD depletion in some daughter cells. However, as transplanted cells *in vivo* ought to discontinue in proliferation and start differentiation, we assume that the division-caused QD label loss will be of less importance in regard to cell tracking purposes *in vivo*. In summary, the here used red-emitting quantum dots emerge as easy to handle, cell friendly label for rat pancreatic stem cells.

## Figures and Tables

**Table 1:** List of the used QuantiTect Primer Assays commercially available from Qiagen.

Target	QuantiTect Primer Assay	Amplicon (bp)	*T*_m_ in °C
*β*-actin	Rn_Actb_1_SG QuantiTect Primer Assay	145	86,20
Nestin	Rn_Nes_1_SG QuantiTect Primer Assay	63	79,60
Kruppel-like factor 4	Rn_Klf4_1_SG QuantiTect Primer Assay	66	83,05
Oct4	Rn_Pou5f1_1_SG Quantitect Primer Assay	134	88,05
CD9	Rn_Cd9_2_SG QuantiTect Primer Assay	124	82,20
GATA-binding protein 4	Rn_Gata4_1_SG QuantiTect Primer Assay	70	81,90
Aggrecan1	Rn_Agc1_1_SG QuantiTect Primer Assay	90	82,15
Osteopontin	Rn_Spp1_1_SG QuantiTect Primer Assay	122	83,45
*α*-smooth muscle actin	Rn_Acta2_1_SG QuantiTect Primer Assay	82	82,90
PPAR*γ*	Rn_Pparg_1_SG QuantiTect Primer Assay	146	82,65
Glial fibrillary acidic protein	Rn_Gfap_1_SG QuantiTect Primer Assay	131	83,75
Myelin basic protein	Rn_Mbp_1_SG QuantiTect Primer Assay	117	83,50
*β*3-Tubulin	Rn_Tubb3_1_SG QuantiTectPrimer Assay	114	85,35
Neurofilament medium	Rn_Nef3_1_SG QuantiTect Primer Assay	112	82,80
Integrin *α* 6	Rn_Itga6_1_SG QuantiTect Primer Assay	70	75,75
Caspase-3	Rn_Casp3_1_SG QuantiTect Primer Assay	61	76,20
